# Promising Advances in Pharmacotherapy for Patients with Spinal Cord Injury—A Review of Studies Performed In Vivo with Modern Drugs

**DOI:** 10.3390/jcm11226685

**Published:** 2022-11-11

**Authors:** Dominika Mech, Katarzyna Korgol, Antonina Kurowska, Bartlomiej Adamski, Malgorzata Miazga, Grazyna Biala, Marta Kruk-Slomka

**Affiliations:** 1 Student Clubs and Organizations, Department of Pharmacology and Pharmacodynamics, Medical University of Lublin, Chodzki 4a Street, 20-093 Lublin, Poland; 2 Student Clubs and Organizations, Department of Pharmacognosy and Pharmaceutical Botany, Medical University of Lublin, Chodzki 1 Street, 20-400 Lublin, Poland; 3 Department of Pharmacology and Pharmacodynamics, Medical University of Lublin, Chodzki 4a Street, 20-093 Lublin, Poland

**Keywords:** spinal cord injury, neuropathic pain, oxidative stress, pharmacotherapy

## Abstract

Spinal cord injury (SCI) is a pathological neurological condition that leads to significant motor dysfunction. It is a condition that occurs as a result of tragic accidents, violent acts, or as a consequence of chronic diseases or degenerative changes. The current treatments for patients with SCI have moderate efficacy. They improve the quality of life of patients, but they are still doomed to long-term disability. In response to the modern directions of research on possible therapeutic methods that allow for the recovery of patients with SCI, a scientific review publication is needed to summarize the recent developments in this topic. The following review is focused on the available pharmacological treatments for SCIs and the problems that patients face depending on the location of the injury. In the following review, the research team describes problems related to spasticity and neuropathic pain; possible therapeutic pathways are also described for neuroprotection and the improvement of neurotransmission within the injured spinal cord, and the review focuses on issues related to oxidative stress.

## 1. Introduction

Spinal cord injury (SCI) is a pathological neurological condition leading to significant motor dysfunction. It is a condition that often occurs as a result of tragic accidents or violent acts (90% of patients), but 10% of cases come as the effects of chronic diseases or degenerative changes. The World Health Organization (WHO) records an increase of approximately 350,000 new SCI patients each year. Physiological spinal cord activity involves the pathways of interaction between astrocytes, neurons, microglia, and oligodendrocytes. Following spinal cord injury, these key interactions between groups of cells are damaged, and their normal functioning is permanently disrupted. Spinal cord injury results in impairment or even loss of natural body functions, including sensory and motor impairments [1]. The failure of CNS components to regenerate is a major cause of disability in SCI patients. Considering all possible pathways, it has been noted that the corticospinal tract (CST) is the most resistant to regeneration [2]. 

**Figure 1:**
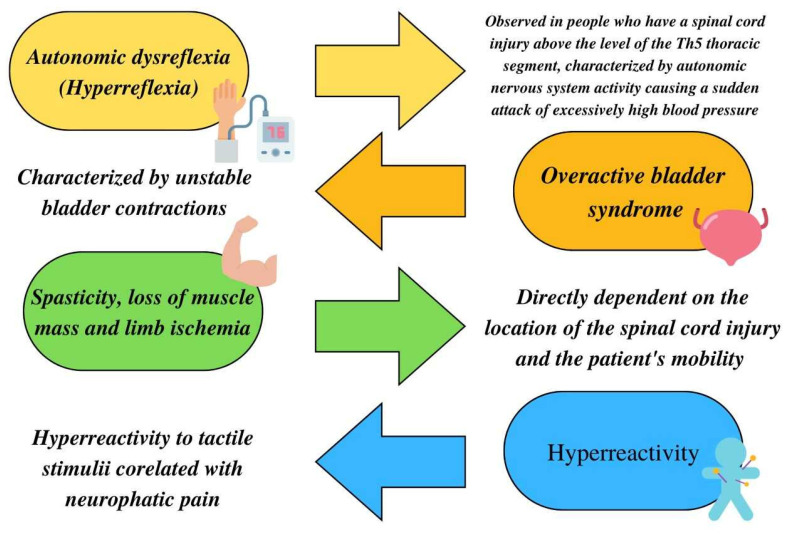
Types of disturbances in tetraplegia.

Patients with quadriplegia (tetraplegia) and biplegia (paraplegia) also face many other types of disorders, as shown below (Figure 1).

Current treatments show moderate therapeutic efficacy, and although they significantly improve the quality and length of life of patients, they still condemn the patients to years of disability. In the last few years, however, an enormous number of scientific publications have appeared describing new scientific findings on the potential possibility of further improving the quality of life of patients with SCI. 

This paper is, on the one hand, an overview of the available pharmacological treatments for SCI and the problems faced by patients who are affected by this condition; on the other hand, this work is also an evaluation of new potential mechanisms of activity of a number of drugs, which in the future may become the leading foundations for the prevention and/or treatment of the effects of SCI. 

## 2. Etiology and Types of SCI 

Spinal cord injury appears mostly as a result of automobile crashes and falls. Other listed causes are: gunshot wounds, motorcycle crashes, diving incidents, and medical/surgical complications [3]. 

To classify an SCI, the International Standards for Neurological Classification of Spinal Cord Injury developed an exam based on three scores. The test consists of the American Spinal Injury Association (ASIA) motor score, which grades muscle strength and movement, the ASIA sensory score, which grades light touch and pinprick feeling, and the ASIA impairment scale, which assigns the severity of the SCI [4]. 

Another tool that can be used to grade SCIs is the Frankel scale; its individual components determine the degree of preserved motor and sensory function. However, the ASIA scale appears to be more reliable [5].

Spinal cord concussion is a phenomenon that mainly occurs during contact sports and minor car accidents [6]. It is characterized by varying degrees of sensory impairment and muscle motor weakness. Spinal concussion is usually resolved within 24–72 h [3]. At the same time, a large group of patients complain of a recurrence of symptoms after treatment has already been completed [6,7]. This is further proven by the indication that adults are burdened with a higher predisposition to this type of injury due to reduced spinal mobility and spinal canal stenosis compared with the pediatric population [8]. 

In clinical practice, there are also the concepts of spinal cord contusion and crush. Contusion has been classified as a more serious case compared with concussion. This injury refers to the destruction of spinal tissue through mechanical damage, bleeding, or compression by bony structures. The changes and dysfunction are contained within a broad framework and are permanent in nature. Similarly, crushing of the spinal cord produces irreversible symptoms of motor impairment below the site of injury. 

Primary SCI also includes injuries resulting from gunshot wounds. Damage in this case may result from the direct impact of the bullet or from the shock effect of the bullet impact and temporary cavitation [9,10]. 

Primary SCI is followed by changes that cause secondary SCI. Secondary cellular changes after the acute phase of SCI, such as dysfunction and cell death, are caused by proapoptotic signaling and ischemic damage that follows the microvascular destruction of the spinal cord [11]. As a consequence of ischemia, the vascular damage also leads to hypoxia. Vascular damage causes extensive hemorrhages that expose the core to an influx of inflammatory cells and cytokines. Increased levels of pro-inflammatory cytokines such as tumor necrosis factor (TNF) and IL-1beta are observed almost immediately after injury; macrophages, neutrophils, and lymphocytes also appear. The body’s inflammatory response becomes so great that swelling of the spinal cord occurs, leading to a mechanical compression of the cord. The mechanical compression created by the emerging swelling and/or bony fragments leads to the deterioration of the patient and an increased severity of the injury [4]. Intraspinal causes of secondary spinal cord injury are explained by Hall and Wolf’s theory [12]. A pharmacological analysis was then performed to determine the possible role of abnormal calcium influx, vasoactive arachidonic acid metabolites, and microvascular lipid peroxidation in the development of white matter ischemia within the spinal cord. The hypothesis put forward concerned the pathogenesis of post-traumatic central nervous system ischemia. It was found that this process significantly affects the increase in intracellular Ca^2+^ ion concentration as well as the increase in the synthesis of vasoactive prostanoids such as prostaglandin F2alpha and TXA2. The process also results in progressive microvascular lipid peroxidation. 

**Figure 2:**
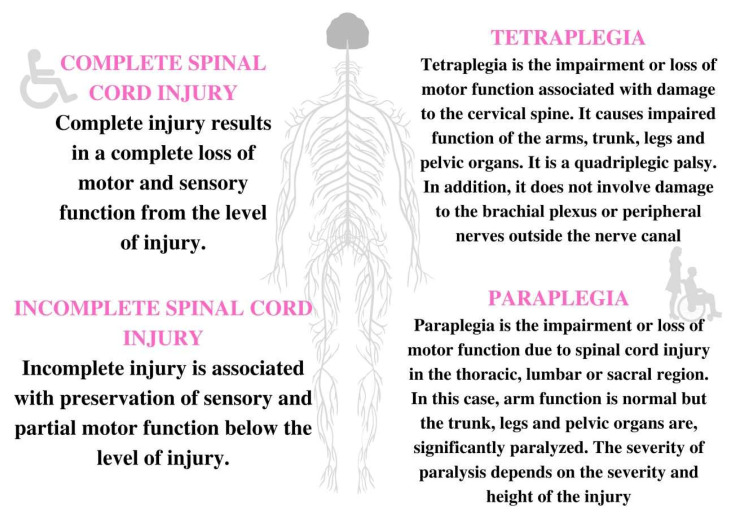
Degrees of SCIs.

There are several degrees of SCI. The types of injury depend on several degrees, which are shown in Figure 2 [13,14]. 

## 3. Inflammation in SCI

SCI is most often caused by mechanical damage (also called primary damage) and the secondary damage that is caused by inflammation [15]. The initial injury triggers successive pathophysiological cascades and activates cellular processes that contribute to secondary tissue damage [16]. The blood–spinal cord barrier is destroyed, which promotes the infiltration of macrophages, neutrophils, and T lymphocytes into the damaged area. Fragments of necrotic nerve cells are removed first. In addition, the cells of the immune system protect against the entry of pathogenic microorganisms [17]. Excessive activity of immune cells leads to the development of inflammation and can lead to a slow degradation of the tissue, subsequently causing the impairment of its function, i.e., functio laesa [16].

Neuroinflammation is an important feature of the CNS response to the occurrence of injury. Moreover, it is one of the factors in the pathomechanism of various neurodegenerative diseases [18]. CNS inflammation can contribute to the long-term death of motor and sensory neurons, and this contributes to an impaired autonomic nervous system function. Despite improvements in primary care, health care, and rehabilitation, the difficulty of treating inflammation after an SCI remains, and this contributes to significant disability and mortality among patients. 

As previously mentioned, SCIs and disabilities are caused by mechanical injury (primary) as well as secondary injury, which contributes to inflammation [18]. The primary phase of inflammation occurs when mechanical pressure on spinal cord tissue can contribute to the destruction of neurons and axons [19]. The incidence and severity of primary injuries can be reduced by increasing safety in the workplace, sports, or recreation, but they cannot be completely prevented. In addition to reducing the incidence of mechanical injuries, it is very important to reduce the extent of secondary injury and eliminate inflammation [18]. The secondary phase of inflammation occurs after the primary phase and is mainly characterized by edema, cavitation, inflammation, apoptosis of nerve cells, and glial scarring [19]. Secondary damage can be reduced by introducing antioxidants, decreasing proinflammatory cytokines, increasing blood supply to the injured tissue site, decreasing cytotoxic glutamate (Glu) levels, and inhibiting the apoptosis of glial cells (e.g., oligodendrocytes) and neurons [18]. It appears that the modulation of harmful inflammation in acute neuritis may be effective in treating it, thereby limiting the injury and allowing the damaged tissues to return to normal function [20]. The primary injury is irreversible, so pharmacotherapy of the secondary injury in SCIs proves to be clinically crucial [21].

Currently, the approach of pharmacotherapy is anti-inflammatory treatment for SCIs. However, the anti-inflammatory drugs known so far do not penetrate the blood–spinal cord barrier, which means that they do not act at the target site of the damage and do not show efficacy. Methylprednisolone, which belongs to the group of glucocorticosteroids, appears to be the most effective, but causes serious side effects [17].

Methylprednisolone was tested for its antioxidant ability to inhibit lipid peroxidation and scavenge peroxynitrite from cell membranes in the NASCIS study that was conducted between 1984 and 1997, at which time the significant therapeutic potential of corticosteroids during SCI therapy was not confirmed. The data presented suggested that efficacy in more than 1500 patients with acute SCI was low, that the treatment did not significantly improve patients’ functional recovery, and all NASCIS studies showed an increased risk of adverse events in the steroid-treated population [22,23]. Publications in subsequent years have confirmed the lack of sufficient evidence for using high doses of methylprednisolone within eight hours after acute spinal cord injury as a treatment guideline [24].

For this reason, there is a need to search for new drugs that are effective in treating inflammation after SCI with minimal side effects [17].

### 3.1. The Role of Microglia

Microglia, especially those located at the periphery of the medulla, also play an important role in the secondary injury after an SCI. Activating microglia negatively affects neuronal function and leads to neuroinflammation, toxicity, and inhibition of neuronal cell growth by producing proinflammatory molecules [15,25].

However, it also has anti-inflammatory and neuroprotective effects to some extent, mainly within damaged nerves after an SCI [15]. Additionally, microglia have the ability to maintain calcium homeostasis and prevent calcium-dependent excitotoxicity. In the treatment of inflammation after an SCI, microglia accelerate the scarring of astrocytes, which prevent immune cells from entering the damaged spinal cord [18]. Depending on the stages of SCI, the role of microglia may change. An important goal of pharmacotherapy is to normalize microglia function to enhance the regeneration of damaged neurons [15].

### 3.2. Bioactive Mediators of Inflammation

In the treatment of inflammation after an SCI, bioactive lipids involved in the moderation of inflammation have also been shown to play an important role. The group of bioactive lipids includes many versatile mediators and regulators of inflammation, which include prostaglandins and the related eicosanoids. These compounds are formed from fatty acids that are bound to the cell membrane, the so-called phospholipids. For eicosanoids to be converted to active mediators of inflammation, the presence of several important enzymes is required, including the important phospholipase A2 (PLA2). On the other hand, PLA2 is responsible for the formation of AA, DHA, EPA acids and lysophospholipids by the same mechanism, which are used for the subsequent synthesis of bioactive lipid mediators with anti-inflammatory effects [26]. Thus, phospholipase A2 is involved in the initiation of inflammation in neurological disorders as well as in the resolution of inflammation. There are a very large number of isoforms of this enzyme, which differ biochemically, structurally, and functionally; this causes some difficulties in treatments. Pharmacotherapy of inflammation after SCI depends on the type and occurrence of cells that are responsible for the secretion of PLA2 enzyme isoforms. In SCIs, there is an imbalance between prostaglandins, leukotrienes, and free fatty acids (PUFA) (arachidonic acid (AA), docosahexaenoic acid (DHA), eicosapentaenoic acid (EPA)), as well as between the amount of free fatty acids alone [18,27]. After SCI, there is an increased level of free AA and decreased DHA at the site of the lesion. Because these two PUFAs have antagonistic effects on inflammation, this imbalance in PUFA homeostasis likely contributes to the exacerbated and chronic inflammatory response that occurs after neuro-injury. Despite the difficulty in treating inflammation after SCI with lipid mediators, there is hope of finding a promising molecular target that will prove effective in pharmacotherapy. Several strategies to increase n-3 PUFA levels after CNS injury have shown beneficial results. One example is supplementation with ALA, EPA, and DHA, which improve neuronal cell regeneration, minimize oligodendrocyte and neuronal loss, and further attenuate inflammation in various mouse and rat models of SCIs [28,29,30].

### 3.3. Chemokine-Receptor Ligand System 

An important aspect of SCI pharmacotherapy is the modulation of inflammation in the injured spinal cord. Chemokines are small molecules that affect the movement of immune cells, causing chemotaxis. In addition, chemokines are involved in T cell growth and differentiation, apoptosis, cell cycle, angiogenesis, and metastatic processes. They can regulate the production of free radicals, nitric oxide, cytokines, and matrix metalloproteases. About 50 chemokine genes are currently known in humans. Due to the wide occurrence of receptor ligands, they are divided into four subfamilies that take into account their chemical structure. Chemokine CCL3 belongs to the group of inflammatory and inducible chemokines, which are regulated by transcriptional mechanisms during inflammation [31,32]. The chemokine ligand receptor system is considered an important factor involved in this inflammatory response. The chemokine CCL3, also called macrophage inflammatory protein (MIP)-1α, is regulated in a transcriptional mechanism during inflammation [33]; it is found in most mature hematopoietic cells, monocytes, macrophages, neutrophils, and in microglia and astrocytes. CCL3 is involved in stimulating immune cells, and its levels significantly increase after an SCI. Antisense oligonucleotides (ASOs) and 2’-deoxy-2-fluoro-D- arabinonucleic acid (FANA), which is an oligonucleotide analog that shows affinity for RNA, have been shown to form a FANA:RNA hybrid, reflecting the structure of the native DNA:RNA hybrid as a chimeric ASO FANA-DN [33,34]. This complex clearly modulates inflammation by suppressing CCL3 expression in the mouse spinal cord, leading to reduced levels of pro-inflammatory cytokines and improved functional recovery after spinal cord injury. This treatment strategy offers hope for improving pathological conditions not only after SCIs but also in other CNS diseases [16].

### 3.4. Heme Oxygenases (HO)

Heme oxygenases (HO) are enzymes that catalyze the breakdown of heme [35]. Heme oxygenase-1 (HO-1) is mainly induced in stress situations compared with heme oxygenase-2 (HO-2), which is constitutively present in cells but can also be induced [36].

Several studies have shown that HO-1 can play a protective role in the early phase of SCI inflammation, that is, when the blood–brain barrier has not yet been breached. In contrast, microglia play an anti-inflammatory and neuroprotective role on damaged core cells in the secondary phase of inflammation after SCI. One study tested the effect of heme oxygenase-1 on the microglia response in a rat model. The results partially confirmed the effect of HO-1 on inhibiting the inflammatory response in which microglia are involved. In addition, HO-1 enzyme alleviated neuroinflammation after SCI. This study raises the possibility of using the transplantation of modified microglia with HO-1 overexpression when treating patients after SCI, which can only have an impact on functional core regeneration [15]. 

### 3.5. Apelin-13

An endogenous neuropeptide otherwise known as apelin is involved in the modulation of inflammation after SCI. Several studies have shown that the mRNA of apelin and its receptor (APJ) are expressed in the different parts of the CNS, such as the thalamus, hypothalamus, amygdala, substantia nigra, pituitary gland, medulla oblongata, and spinal cord [37,38]. Apelin mRNA and its receptor (APJ) have been shown to be expressed at various sites in the CNS. Additionally, this peptide is found in the cell bodies of neurons and oligodendrocytes. Apelin and its receptor APJ have beneficial effects on neuronal function by increasing neuronal survival and improving nerve conduction. It has been documented that apelin levels are significantly altered in pathological conditions of the central nervous system such as Alzheimer’s, Parkinson’s, and Huntington’s disease [37,38]. Studies have also shown an indirect reduction in the inflammatory process through the use of apelin-13, which inhibits the release of pro-inflammatory cytokines (PICs) such as IL-1β, IL6, and TNF-α [39], which are involved in the inflammation of spinal cord injury; apelin-13 also increases the levels of anti-inflammatory cytokines such as IL-10. In addition, apelin-13 may have the effect of increasing the volume of the medulla and increasing the number of nerve cells in that location. Thus, apelin shows neuroprotective effects and has become another new pharmacotherapy target for the treatment of inflammation after SCI and spinal cord reconstruction [19].

### 3.6. Nanotherapeutics

Nanotherapeutics are drugs combined with polymers to enhance the ability to target the drug to the affected area. These polymers exhibit biocompatibility with the diseased tissue, making treatment more effective. Such polymers include poly(lactic-co-glycolic acid) (PLAG), poly(ethyleneimine) (PEI) and methoxypolyethylene glycol (mPEG) [40]. More and more studies suggest the efficacy of this nanoparticle-based drug delivery system; as has been found, they are promising strategies for regulating inflammation [41]. Following SCI injury, hemorrhage and ischemia occur at the site of blood–spinal cord barrier damage. Immune cells such as macrophages also accumulate there. M1 macrophages are mainly responsible for inflammation, releasing inflammatory factors and reactive oxygen species (ROS) that lead to permanent tissue damage. The opposite is true for M2 macrophages, as they enhance axonal activity and repair of motor function. During blood–spinal cord barrier destruction, increased expression of matrix metalloproteinases (MMPs) is also observed, which exacerbates the blood–spinal cord barrier destruction [17]. An example of MMP-responsive molecules is activated cell-penetrating peptides (ACPPs). Hence, ACPP-modified nanoparticles have emerged as ideal nanocarriers that target damaged tissue across the damaged barrier. A biocompatible nanocarrier delivery system targeting the injured spinal cord was developed. This system is composed of PLAG, PEI, mPEG polymers to form a triblock comolymer (PPP), and an MMP-targeting peptide (ACPP) synthesizing the PPP-ACPP structure to load the anti-inflammatory drug, which is the TNF-α blocker etanercept (ET), to form the nanotherapeutic ET@PPP-ACPP. The main mechanism of the drug is to inhibit the secretion of TNF-α factor. Moreover, it is endocytosed and degraded by macrophages in the damaged tissue. It affects the regulation of the NF-κB signaling pathway, which is responsible for the transformation of M1 to M2 macrophages and polarizes M2 macrophages, resulting in a decreased secondary production of pro-inflammatory cytokines and increased production of anti-inflammatory cytokines. ET@PPP-ACPP has been shown to accumulate in the altered area of damaged tissue and achieve effective treatment of SCI. Additionally, in a rat model, promotion of locomotor regeneration was demonstrated [17]. 

### 3.7. Ferulic Acid (FA)

In secondary injury after SCI, in addition to the inflammatory response, apoptosis and cellular autophagy also play an important role, contributing to neuronal destruction [21]. Autophagy, also called autophagocytosis (literally meaning “self-eating”), is a natural process of gaining additional energy by breaking down cellular particles, fragments, or organelles. This allows cells to differentiate and maintain homeostasis. Thus, enhancing autophagy can alleviate SCI in rats, resulting in a return to neurological function. Additionally, it has been demonstrated that autophagy can arrest enhanced apoptosis and exhibit protective effects on neurons [42]. An important regulator of autophagic pathways is Beclin 1, whose participation was found in SCI pathology. Apoptosis of nerve cells is influenced by the factors Bax and Bcl-2. Modulating the above factors associated with inflammation (mainly cytokines), apoptosis (Bax and Bcl-2), and cellular autophagy (Beclin-1) may be beneficial in reducing secondary damage in SCI [21].

FA is a derivative of cinnamic acid that is naturally found in cereals: rye, wheat, oats, but also in nuts and coffee beans, among others. When FA was tested for SCI in a rat model, it showed several important actions. FA in a behavioral study protected rats from SCI-induced motor dysfunction. The compound clearly has a neuroprotective effect, as tissue regeneration of spinal cord neurons was noted after 28 days. With chronic use of FA, a decrease in the expression of the inflammatory factors IL-1β, IL-6, and TNF-α was observed through the upregulation of NF-κB in the spinal cord. Furthermore, a decrease in the expression of COX-2 and iNOS enzymes was noted. Another important aspect is the degree of apoptosis, which is determined by the Bcl-2/Bax ratio. A decreased expression of Bcl-2 and increased dominant factor (Bax) can stimulate cell apoptosis in the injured core by SCI. After FA treatment, this ratio was reversed, confirming the efficacy of neuronal neuroprotection and thereby reducing cell apoptosis [21]. 

### 3.8. Neuroinflammation 

Neuroinflammation is mainly observed in the secondary phase of SCI. Activating microglia are produced with an increase in proinflammatory cytokines. A cascade of signaling pathways leads to an increase in numerous proinflammatory factors, and free radicals contribute to exacerbate secondary damage [43].

Mitogen-activated protein kinase (MAPK): it is now known that the ROS and MAPK signaling pathway can affect the activation of nuclear factor κB (NF-κB), which is an essential element involved in neuroinflammation and is activated by microglia after SCI. Inhibition of the NF-κB signaling pathway results in a decreased expression of inflammatory factors important for inflammation after SCI. These include IL-6, TNF- α, and IL-1β, among others. This fact favors pharmacotherapy of secondary damage caused by microglia-mediated inflammation [44]. 

Kaempferol: this compound plays an important role in neuroinflammation. This compound is one of the natural polyphenols most commonly found in tea, broccoli, grapefruit, kale, and cabbage. Kaempferol is a flavonoid with anti-inflammatory and antioxidant properties. In studies, kaempferol has been shown to alleviate oxidative stress mediated by microglia activation in the secondary phase of SCI. In addition, this flavonoid promoted recovery of front limb motor function in a rat model of SCI. Referring to the MAPKs responsible for inflammation, kaempferol was shown to inhibit the activation of cascade proteins and decrease the activity of the ROS-dependent MAPK-NF-κB signaling pathway, thereby reducing the levels of pro-inflammatory factors such as IL-1β, TNF-α, and iNOS in microglia. In addition, this compound inhibits the activation of the NLRP3 inflammasome-related pyroptosis pathway. This study showed that kaempferol may contribute to the reduction of oxidative stress and neuroinflammation. To date, this is the sole and primary pathophysiological mechanism that has been identified with respect to kaempferol’s neuroprotection in SCI [43].

## 4. Oxidative Stress and Neuropathic Pain in SCI

ROS are molecules containing at least one unpaired electron and seek to exchange electrons between other molecules; hence, they are highly reactive. The main generator of free radicals in the body is the respiratory chain (90%). The remainder are physiological reactions occurring in various cells of the body. Adverse processes involved in the formation of ROS are (among others): the effects on the cell of, for example, ultraviolet radiation; ionizing radiation; increased temperature; and mechanical pressure (the so-called injury). Free radicals play an important role in many life processes; they participate in the regulation of gene expression, protein phosphorylation, and calcium concentration in cells. They participate in cell division and the elimination of microorganisms. Excess of free radicals is harmful, leading to destruction of cell structure and function through apoptosis or necrosis [45]. In SCIs, oxidative stress is important in addition to inflammation. Oxidative stress is characteristic of the secondary phase of SCI. Together with vasospasm, decreased pressure, worsening ischemia, excitotoxicity, and inflammation, oxidative stress significantly contributes to irreversible damage to cells and surrounding tissues, causing pain and loss of function. Studies show that the most common syndrome of SCI is characteristic neuropathic pain (NP), which significantly impair the patient’s quality of life [46,47].

### 4.1. The Causes of NP in SCI

Pain stimuli trigger a cascade of responses that lead to the activation of N-methyl-D-aspartate (NMDA) receptors. Sensitization of these receptors promotes an increased influx of Ca^2+^ into the cell, which triggers intracellular signaling pathways, thereby enhancing the nociceptive response. Overactivation of the glutaminergic system is associated with abnormalities in the sensory system (peripheral and central), stimulating neurons and causing abnormal pain (intrinsic pain, hyperalgesia, allodynia) [46,47]. Furthermore, the formation of ROS—particularly those such as superoxide (O2-), hydrogen peroxide (H2O2), nitric oxide (NO), and peroxynitrite (ONOO-)—and the formation of non-hydrogenated molecules such as hydrogen peroxide (H2O2) significantly contribute to neuronal damage and CNS cell loss, particularly after injury and ischemia [46,47,48]. ROS at the subcellular and cellular level have been shown to be one of the major contributors to NP formation [46,47].

A clear association was found between the development of neuropathic pain and the presence of oxidative stress and hyperactivity of NMDA receptors for Glu [46,47].

### 4.2. Pharmacotherapy NP

Treatment of NP remains one of the challenges of medicine, as commonly used painkillers do not relieve the pain. Treatment of NP usually starts with weak analgesics, mainly from the group of non-steroidal anti-inflammatory drugs (NSAIDs); weak opioids are prescribed if the pain is severe. When the mentioned drugs are ineffective, treatment with strong opioids (e.g., morphine) or coanalgesics (including tricyclic antidepressants and anticonvulsants) is implemented. This type of treatment is not so effective and is associated with many side effects. Thus, there is a need to search for new drugs with higher pharmacotherapy effectiveness and lower harm caused by side effects [46,47]. The aim of modern pharmacotherapy in the alleviation of neuropathic pain, hyperalgesia, and allodynia is the introduction of substances with different mechanisms of action, including: inhibitors of the glutamatergic system, such as NMDA receptor antagonists and antioxidants, which have been used to prevent the formation of free radicals contributing to their reduction and protection of neurons; and compounds modifying genetic pathways.

### 4.3. Groups of Drugs with Potential Use in the Treatment/Control of NP in SCI

#### 4.3.1. Coanalgesics

Pregabalin is used as the drug of choice in SCIs [49]. It works by attaching to the α2δ-1 subunit of the voltage-activated calcium channel, resulting in the relief of neuropathic pain. Scientists believe that the α2δ-1 subunit has a significant influence on the development of this pain. Researchers have also noted that pregabalin also binds to the α2δ-2 subunit, which is mainly located on inhibitory neurons. Therefore, it has been speculated that pregabalin has a different effect on each of these subunits [50]. In the treatment of SCI, this drug is considered to be one of the better-acting drugs in NP. It has been shown to be the most effective in reducing pain among the group of coanalgesics such as gabapentin, carbamazepine, and amitriptyline. It is effective and safe; however, pregabalin more often causes side effects (including somnolence and nausea), which can lead to the discontinuation of the drug [49,50,51]. This drug can also be addictive and can be abused due to the euphoria and improvement of social functions in higher doses. Intoxicating, activating, sedative, dissociative, or amnestic effects have also been reported. It is also used to increase the level of sensations while taking other stimulants, such as alcohol or opioids [52].

Gabapentin has a mechanism of action that is similar to that of pregabalin. This drug binds with the α2δ-1 subunit of the voltage-activated calcium channels [53]. Its potency is similar to that of pregabalin, but gabapentin reduces the pain level to a lesser extent. Gabapentin is characterized by the lowest side effects in the group of the aforementioned coanalgesics, which may turn out to be extremely important from the perspective of a patient who is particularly sensitive to the side effects of these drugs [49,51]. The drug, similarly to pregabalin, may cause addiction [52]. It has been shown that gabapentin at doses of 1800–3600 mg can provide good results in relieving neuropathic pain. There is evidence for its activity within the relief of postherpetic neuralgia and diabetic peripheral neuropathy, while evidence for other types of neuropathic pain is limited. Approximately 30–40% of study participants achieved a reduction in pain severity by at least half after gabapentin therapy, compared with the 10–20% in the placebo group; this is a significant difference. The beneficial effects of gabapentin on reducing sleep disturbances, fatigue, and depression must also be considered. It should also be noted that as many as more than 50% of people treated with gabapentin do not experience pain relief, but experience the side effects [54]. At the same time, the number of negative studies has increased over the past few years. The analgesic effect of gabapentin is associated with a reduction in central sensitization through binding to the α2δ subunit of calcium voltage-gated channels [55]. In the treatment of peripheral neuropathic pain, favorable results have been obtained by replacing high doses of monotherapy with combination treatment via tricyclic antidepressants or an opioid [56,57,58]. These studies provide justification for the use of moderate-dose medications in patients who can poorly tolerate high doses of gabapentin monotherapy. 

Tizanidine is a drug commonly used in the treatment of spasticity; however, animal studies have shown that it can reduce NP [59]. The drug is an α2 adrenergic receptor agonist, and although its mechanism of action is not fully understood, there is a noticeable reduction in TLR4/NF-κB (Toll-like receptor 4/nuclear factor κB) signaling, which reduces the levels of pro-inflammatory cytokines. In SCI, this enhances the signaling and enhances the production of cytokines, including IL-1β, IL-6, and TNF-α. Thus, it can be considered that this molecular target may play a major role in the occurrence of neuropathic pain (Tizanidine exerts antinociceptive effects in the spared nerve injury model of neuropathic pain through an inhibition of TLR4/NF-κB pathway). Moreover, a clinical trial in 2000 confirmed that pain was reduced or completely absent in the human NP population after administration of tizanidine. The researchers also pointed out the side effects experienced by patients, including somnolence or insomnia, dry mouth, and gastrointestinal disorders, and the need to exclude patients from therapy who had elevated liver function tests [60].

#### 4.3.2. Cannabinoids

These are substances that act through an agonistic effect on cannabinoid (CB) receptors, i.e., type CB1 receptors found in the central and peripheral nervous systems and CB2 receptors mainly found in the immune system. Cannabinoids also modulate the action of many other neurotransmitter systems. Cannabinoids are more and more often used in many ailments; therefore, they are also sought for in ailments related to SCI because they can tolerate spasticity and reduce pain, including inflammatory pain. In preclinical studies, it has been observed that cannabinoids modulate the action of neurotransmitters by inhibiting the release of neurotransmitters and neuropeptides from presynaptic neurons, suppress nerve inflammation, and relieve pain by activating descending pain-inhibiting pathways. Scientists indicate that this action may be caused by an inverse agonism to the orphan receptors GPR3, GPR6, and GPR12, which when activated trigger neuropathic pain but also stimulate cell proliferation and are responsible for the growth of neurons. Moreover, after administration of CBD, a decrease was noticed in the concentration of prostaglandin E2, malondialdehyde, and nitric oxide, which are involved in nociception [61].

A study on patients with neuropathic pain showed a reduction in neuropathic pain after using Δ9-tetrahydrocannabinol (THC) or a mixture of THC with cannabidiol (CBD). Cigarettes with THC (3.5% and 6.9% THC), vaporization with THC (2.9% and 6.7% THC), and sublingual sprays with a lot of CBD and THC were used in the study. The much better antinociceptive effect of THC compared with CBD is also noticeable, but due to the possibility of addiction and the range of side effects of THC, CBD seems to be a better drug in the treatment of neuropathic pain. It should be mentioned, however, that some studies have not shown an antinociceptive effect in the treatment of neuropathic pain with either CBD or THC [48].

In addition to the beneficial effects of cannabinoids, it is imperative to mention the potential risks associated with their use. The most common side effects include fatigue, diarrhea, nausea, hepatotoxicity, and drug-induced drowsiness and sedation [62,63,64]. These effects are dose-dependent and add up after the administration of other central nervous system-inhibiting drugs (alcohol, antiepileptic drugs). Chronic cannabis use is also associated with the risk of cravings after abstinence. Regular cannabis users have been shown to exhibit moderate to high responses to cannabis-related cues, and cannabis activates the brain reward pathway more than natural rewards [65,66].

#### 4.3.3. NMDA Receptor Antagonists

Amantadine is a well-known NMDA receptor antagonist and has found use as an antiviral drug and in the treatment of dyskinesias in Parkinson’s disease. In recent studies, the drug has been tested against the alleviation of oxidative stress and reduction of excitotoxicity caused by an overactivation of the glutamatergic system in NP after SCI. Other studies have shown that the use of NMDA antagonists including ketamine and dextromethorphan significantly reduce neuropathic pain sensation. Amantadine was shown to induce antinociceptive effects in rats with SCI after immediate administration, and these effects were regulated by the oxidative stress and excitotoxicity present. Importantly, amantadine appears to be safe and when chronically administered at low doses causes few adverse effects [47]. Pharmacotherapy with amantadine may offer promising prospects in alleviating NP in patients after SCI; however, more studies are needed to substantiate this.

More and more studies confirm that the use of NMDA receptor antagonists also reduces allodynia and hyperalgesia [46]. To date, dapsone has been used as an antimicrobial drug that inhibits folic acid synthesis. In recent SCI studies, the drug has demonstrated antioxidant, anti-inflammatory, anti-apoptotic, and anti-excitotoxic activities [46]. When administered at an acute dose in a rat model, it reduced tactile allodynia (TA) and mechanical hyperalgesia (MH) with superior results and effect compared with the use of gabapentin. The mechanism of action of dapsone involves the blocking of receptors for Glu through the inhibition of its agonists kainic acid and quinolinic acid [46,47]. Thiol compounds are a naturally occurring reservoir in living cells, and studies have shown reduced glutathione (GSH) levels in nerves and spinal cord in rats with neuropathic pain [32]. An imbalance between the GSH levels and lipid peroxidation (LP) results in oxidative stress [46,47]. Increased free radical generation affects sensory hypersensitivity, and this may contribute to the development of NP after SCI. Acute administration of dapsone reduces TA and MH induced by ROS in SCI in rats. The antioxidant effect of dapsone is the reduction of lipid peroxidation induced by free radicals generated at the injury site. In NP, there is an increased production of NO by nitric oxide synthase (nNOS) in the CNS associated with an overactivity of the glutaminergic system and influx of calcium ions into the cell. The presence of increased NO contributes to the development of NP [46,47]. The neuroprotective effect of dapsone is related to its ability to reduce glutamate-modulated excitotoxicity and oxygen and nitrogen radical levels. This drug is a neuroprotective agent in the treatment of ischemia and epilepsy. Our results indicate that dapsone may exhibit a clear and effective neuroprotective effect in the treatment of SCI and contribute to the elimination of neuropathic pain that appears immediately after injury [47].

#### 4.3.4. Antioxidants

To date, studies have found that vitamin D plays an important role in regulating reactive oxygen species levels. Vitamin D participates in the expression of antioxidant systems, which reduce oxidative stress by scavenging free radical molecules and reversing oxidative changes produced during ROS signaling. Reducing ROS levels significantly attenuates the pain responses favored during NP. Recent meta-analyses of clinical trials have shown that vitamin D supplementation increases total antioxidant capacity along with GSH levels and reduces ROS-induced blood lipid peroxidation [48,67]. These properties led researchers to administer an active form of vitamin D3 to rats with chronic compression injury (CCI) to the sciatic nerve and spinal cord with CCI-induced neuropathic pain [48]. The rat model stimulated symptoms of chronic nerve compression that corresponded to the complex regional pain syndrome found in humans. Vitamin D3 administration attenuated the threshold of mechanical withdrawal and thermal latency (as an indicator of antinociception) induced by CCI in the rat model [48]. The mechanism of action of the active form of vitamin D3 includes reducing the levels of hydrogen peroxides and lipid hydroperoxides in the injured sciatic nerve. Vitamin D decreases the activity of nicotinamide adenine dinucleotide phosphate oxidases (NOX), which generate ROS. In addition, vitamin D up-regulates superoxide dismutase and glutathione peroxidase expression. This contributes to increased GSH levels and decreased LP induced by oxygen free radicals [48]. In addition, vitamin D3 administration also increases glucose-6-phosphate dehydrogenase activity, which contributes to increased GSH levels [48]. The above evidence for the antioxidant effect of vitamin D may be used in further studies for oxidative stress-related diseases. Vitamin D3 supplementation may reduce tissue damage and neuropathic pain caused mainly by oxygen and nitrogen free radicals, which provides new opportunities for the pharmacotherapy of inflammation and NP occurring after SCI, among other applications.

Many types of pain are usually relieved by ozone therapy, which has a potential neurotoxicity with a mechanism of action that is not fully understood. Ozone is a gas with strong oxidizing properties depending on its concentration. At increased ozone concentrations, negative effects on the homeostasis of the redox system are observed. These changes mainly occur within the central nervous system due to its strong dependence on oxygen. Overuse of ozone usually leads to neuronal damage [68]. Applied medical ozone forms a mixture with oxygen. In the treatment of various diseases, ozone can interact with organic molecules, leading to the production of reactive oxygen species (ROS) and lipid oxidation products (LOPs) [68]. Previous studies have reported that ozone activates stress-dependent calcium ion release from the endoplasmic reticulum and the calmodulin-dependent protein kinase II/MAPK signaling pathway, contributing to spinal cord neuron (SCN) neurotoxicity. The autophagy signaling pathway as well as the nuclear factor (erythroid-2-derived)/antioxidant response element (NRF2/ARE) play a key role in the antioxidant system [69]. The NRF2/ARE pathway is an important antioxidant stress signaling pathway and is involved in all neuronal system processes as well as normal nervous system activity. Autophagy is a major intracellular degradation system and protects cells from damage, especially in diseases of the nervous system. The p62 protein is a key protein found in autophagy. Autophagy capacity is enhanced by increasing LC3II and decreasing p62 protein levels; the opposite is true when autophagic capacity is impaired. In addition, p62 protein with NRF2 has been shown to form a positive feedback loop, increasing the antioxidant capacity of cells to protect them [70]. A decrease in p62 protein and LC3II levels is observed after ozone overdose, indicating that autophagic flow is impaired. Application of tert-butylhydroquinone (tBHQ) inhibited the decrease in p62 protein and LC3II levels, indicating activation of the autophagy process. The increase in p62 protein expression may have simultaneously resulted from the activation of autophagy as well as the NRF2/ARE pathway after ozone overdose and tBHQ administration. We found that tBHQ activates autophagy and the p62/NRF2/ARE pathway, which indirectly increases the concentration of NRF2 factor in nuclei and enhances the antioxidant system. This mechanism may protect spinal cord cells; however, evidence is still lacking regarding the exact functions of tBHQ in SCN damage due to ozone overdose [68]. The NRF2/ARE pathway and the p62 and LC3II proteins may show an important role as novel antioxidants to treat the oxidative stress occurring after SCI and inhibit ROS-induced tissue damage. It is possible that they will become a new target for SCI research.

Currently, emergency treatment after SCI includes drugs from the group of antioxidants, blockers for neurotransmitters, phosphokinase stimulators, and phosphatase inhibitors. Unfortunately, these drugs do not affect the direct cause of damaged nerves, so the object of research has become alpha-lipoic acid (ALA) [70]. ALA is an eight-carbon saturated fatty acid that is continuously found in the human body. To a small extent, it is spontaneously produced during metabolism, and in larger amounts it is supplied with food. ALA has two important functions: as a coenzyme, it participates in metabolic pathways; and as an antioxidant, it has strong antioxidant properties [70]. To date, ALA has been shown in rat model studies to improve intrarenal blood flow, increase physiological antioxidant levels, and reduce oxygen free radicals in the diabetic nerve. Nerve damage in the spinal cord is caused by, among other things, oxidative stress and oxygen free radicals, whose levels in SCI are above normal. Natural antioxidant substances are unable to inhibit the worsening hypoxia and neuronal destruction. Therefore, ALA was used, and when administered in rats after SCI, the acid increased the effectiveness of other antioxidants, resulting in lower levels of free radicals and oxidative stress [70]. The reduction of negative oxygen balance significantly improves cell and tissue oxygenation and leads to a reduction of free radicals which reduces damage, thus leading to improved neuronal function. This property may prove to be successful in patients suffering from polyneuropathies caused by oxidative stress after SCI mainly occurring during the secondary phase. 

#### 4.3.5. Other Modification of Genetic Pathways 

Histone deacetylase inhibitors (HDAC inhibitor) and inhibitors of the bromodomain protein and extra-terminal domain (BET inhibitor)—it has been noticed and confirmed by research that histone acetylation has a significant impact on the occurrence of chronic pain. Histone acetylation consists of adding an acetyl group to the amino terminus of lysine with the participation of histone acetyltransferase (HAT), while the reverse process, deacetylation, is performed by histone deacetylase (HDAC). While HAT causes chromatin relaxation, which facilitates the transcription process, HDAC causes chromatin condensation, which contributes to the inhibition of transcription. Nerve damage has been observed to lead to an increase in HDAC activity. Hence, HDAC inhibitors have become a potential drug with a proven analgesic effect in the treatment of chronic pain. It has been shown in a mouse experimental model that the HDAC inhibitor SAHA after intranasal administration may even have comparable efficacy to that of pregabalin [20]. The research also investigated the intranasal administration of BET inhibitor BET762, which interacts with so-called “readers” or BET proteins that recognize and bind acetylated lysine residues on histones. Its effectiveness has been confirmed in the treatment of neuropathic pain [20]. Importantly, both drugs reduced the severity of allodynia in the animal model as evidenced by the reduction of thermal and mechanical hypersensitivity in mice. Additionally, after combining two drugs, antiallodynic activity increased [20].

miRNA-139-5p agomir—one of the more recent studies has shown that the use of miRNA-139-5p, the expression of which is significantly reduced in injured spinal cords in a mouse experimental model, results in NP inhibition. Administration of miRNA-139-5p reduces the intensity of neuropathic pain by inhibiting the expression of Mst-1 (Mammalian sterile 20-like kinase 1), which is considered to be one of the particles that may be responsible for the occurrence of neuropathic pain. Its inhibition, in turn, activates the AMPK pathway (adenosine monophosphate-activated protein kinase alpha), which is responsible for shifting the AMP:ATP ratio in favor of AMP. It has been noticed that this activation leads to an inhibition of ROS-induced cell death and inhibition of local inflammation after SCI by inhibiting the activation of NF-κB (Toll-like receptor 4 / nuclear factor κB) and lowering the concentrations of TNF-α (tumor necrosis factor) and IL-1β (interleukin 1β), which are responsible for the inflammatory process. Each of these components of the drug’s action can reduce pain after SCI and at the same time inhibit the progression of neuronal damage [70].

The lncRNA PVT1/miR-186-5p/CXCL13/CXCR5 axis (Long non-coding RNA PVT1/miR-186-5p/chemokine ligand 13/chemokine receptor 5) plays an important role in NP. It was noticed that in the spinal cord of SCI rats, the lncRNA levels of PVT1, CXCL13, and CXCR5 were increased, while miR-186-5p’s levels were decreased. It has also been observed that inhibition of PVT1 lncRNA and long non-coding RNA chains that significantly affect gene expression leads to an increase in miR-186-5p and a decrease in CXCL13/CXCR5, which significantly alleviates neuropathic pain. The decrease in the concentration of PVT1 lncRNA simultaneously leads to a decrease in the activation of astrocytes and a decrease in the expression of neuroinflammatory factors and proteins, which is related to the inhibition of the activity of CXCL13/CXCR5, which is an important regulator of the inflammatory response in the peripheral and central nervous systems. Researchers also indicated that increasing the miR-186-5p level may have the opposite effect compared with the pain-inducing lncRNAs of PVT1, CXCL13, and CXCR5. The study suggests that it can be a new therapeutic target for people with SCI [71].

L-corydalmine is a natural molecule that inhibits the formation of core D1 and D2 dopaminergic receptor complexes. After intrathecal administration of the compound to a rat, a decrease in intracellular calcium concentration and NP levels was observed. A similar effect was noted after administration of Gαq (Gq alpha subunit), PLC and IP3 (inositol triphosphate) inhibitors, and D1 and D2 dopaminergic receptor antagonists, and the administration of their agonists caused pain recurrence. The inhibition of the discussed complex also led to a reduction in the expression of p-PKC γ (protein kinase C γ), p-CaMKII (calcium and calmodulin II dependent kinase), p-CREB (AMP response element-binding protein), and p-MAPK (protein kinase activated by mitogen). Hence, the researchers suggest that the DDR1 and DDR2 receptor complexes can couple to Gαq to increase neuronal excitability via PKC γ, CaMKII, MAPK, and CREB signaling in rat spinal cords. In addition, a study published in 2021 noted that peroxides and the pCamKII pathway contribute to the occurrence of neuropathic pain and that the administration of the CamKII KN-93 inhibitor or free radical scavenger (tempol) reduces mechanical hypersensitivity. Consequently, the discussed molecular target may become a new tool in the treatment of neuropathic pain [72,73].

Inhibition of Rho-related coiled-coil protein kinase—recent studies suggest that SCI is associated with increased p38 phosphorylation in the microglia of the spinal cord and is believed to be one of the main causes of neuropathic pain. Studies report that the activation of p38 MAPK maintains this pain, and that the RhoA/Rho-containing coiled-coil protein kinase (ROCK) pathway mediates p38 activation in the spinal microglia in peripheral nerve damage. The authors suggest that modulation of ROCK signaling may be the subject of a new treatment of neuropathic pain after SCI [74].

## 5. Neuro-Regeneration, Neuroprotection, and Spinal Cord Plasticity

The last two decades of the 20th century saw a tremendous increase in interest and development of research on spinal cord neuro-regeneration. Primary damage to the spinal cord causes secondary damage, which leads to excitotoxicity caused by high accumulation of calcium ions. There is an increase in the concentration of reactive oxygen species and damage to nucleic acids [75]. In the secondary phase, the invasion of monocytes, neutrophils, and T and B lymphocytes and macrophages becomes more intense. Inflammatory cytokines such as interleukin (IL) (IL-1a, IL-1b, IL-6) and TNF-alpha are also released. The infiltration of cells responsible for the immune response promotes inflammation [76]. The following presented research is based on the potential for neuro-regeneration and protection of already existing neural cells from toxic changes caused by primary and secondary SCI. 

### 5.1. Biological Therapy

The first noteworthy study is based on gene therapy as a potential point of resolution for SCI therapy. The cytokines used were hyperinterleukin-6 (hIL-6), which is composed of the bioactive part of the IL-6 protein fused to the alpha subunit of the IL-6 receptor 18 [77]. All experiments used female and male mice that had undergone complete spinal cord crush (SCC) surgery. The mice were administered the above treatment after injury, and significant improvements in functional motility were observed. It was shown that after severe SCC, a single unilateral docking injection of the studied cytokines into the sensorimotor cortex promoted the regeneration of axons and serotonergic fibers of the lumbosacral segments, thus allowing for locomotion of both hind limbs. Transneuronal stimulation of brainstem-localized neurons with molecules may be a promising strategy for achieving functional repair in the human CNS [77,78]. 

In 2021, Fouad et al. published a paper on the effect of the intrathecal administration of the TrkB agonist antibody 29D7 on plasticity after SCI in the cervical region in adult rats [79]. Stimulation of the TrkB receptor caused a cascade of events that are growth signals for the cells. Currently, such therapies are being tested at multiple levels for activity in CNS diseases. Administration of 29D7 to test animals for 4 weeks resulted in as much as a 50% increase in the sprouting of severed corticospinal tract fibers compared with the control group. Increased branching of the grey matter was also observed. Signs of increased plasticity were paralleled by improved paw movement performance. This therapy may be a potential candidate for promoting plasticity and recovery after moderate SCIs. At the same time, the authors emphasize that further studies are needed to confirm the above data. Therapy with 29D7 could be correlated with rehabilitation training and combination therapy in the future.

Another experiment investigated the effects of new chemical compounds on neuroprotective activity. The chemical structure combined *imatinib, a protein tyrosine kinase inhibitor used to treat leukemia*, and 1,3,5-triazines [80]. The choke point of the synthetic derivatives appeared to be nuclear factor kappa B (NF-kappaB), which controls inflammation, proliferation, and cell death. Scientific evidence suggests that inhibiting NF-kB activation provides significant benefits, as it can nullify free radical formation. [80]. One structure was tested in a rat animal model in animals with induced SCI; it was found that the tested derivative improved the motor functions of the rats and also reduced the inflammation and swelling of the spinal cord. A reduction in oxidative stress and inflammation was demonstrated. In a Western blot study, the compound inhibited NF-kB in proportion to the dose.

After SCI, glial scar formation is common, which inhibits axon regeneration and provides a specific barrier to neurotransmission. The next study had its focus on anti-integrin B1 (B1Ab) antibodies [81]. The antibody against integrin B1 (B1Ab) had a therapeutic effect on astrocytes by preventing the induction of scar formation. A study in a mouse animal model was performed, where it was found that TNF-alpha expression in microglia and glial scar was suppressed. The change was attributed to fibronectin, which is a direct ligand of the B1 integrin receptor. The results suggest that B1Ab administration has therapeutic potential in alleviating glial scar formation and persistent neuroinflammation in the chronic phase after SCI. 

Another publication describes the effect of combined intrathecal/intravenous injection of bone marrow-derived stem cells in platelet-rich plasma on SCI in animals [82]. The results showed that the transplanted cells also led to the restoration of spinal reflexes. The first effects were observed 15 days after the procedure. All animals also had their urinary control restored. In one case, the animal showed complete recovery after 90 days of observation. The higher the level of lameness the animals showed, the higher the therapeutic effect was obtained. 

### 5.2. Phosphodiesterase Inhibitors 

Another study investigated the effect of the phosphodiesterase III (PDE-III) inhibitor milrinone, a recommended treatment for heart failure, on experimental SCI [83]. In this case, the study was conducted on 36 rats that were randomly divided into 4 groups, with one group receiving milrinone after SCI. Neurological findings were significantly better in the milrinone-treated group compared with the group that received saline. There were increased levels of glutathione peroxidase in serum and spinal cord tissue. In addition, the levels of 8-hydroxyguanosine, IL-10, and IL-6 were successfully reduced. This clearly indicates a reduced level of inflammation. In the context of neuroprotection, a significant decrease in apoptosis rate was also demonstrated in the study group compared with the control group. On the basis of milrinone studies, it can be concluded that this substance has anti-inflammatory and antioxidant effects and especially reduces apoptosis [83,84]. It thus plays a protective role in secondary SCI.

### 5.3. Immunosuppressive Therapy

Another scientific publication studied the effect of *azathioprine, a potent immunosuppressive and cytostatic drug*, on the course of changes in the body of animals after SCI [85]. Factors such as lipid peroxidation and glutathione peroxidation were taken into account to assess the damage and impact of the test substance. The level of lipid peroxidation was evaluated by measuring the thiobarbituric acid-malondialdehyde (MDA) complexes. Simultaneously, protein carbonyl (PC) levels were measured to determine oxidative protein damage. It was found that the MDA levels in the post-injury groups were significantly higher than those in the azathioprine-treated group. This index was the lowest in the test group compared with all groups. An analogous result was obtained for the protein carbonyl (PC) index. The treatment group had significantly reduced PC levels compared with the injury group. Moreover, the levels of reduced glutathione (rGSH) were significantly higher than the trauma groups, which also shows the positive effect of azathioprine in SCI therapy. 

### 5.4. RhoA/ROCK Inhibitors

Undoubtedly, one of the most valued potential drugs in the field of SCI are the RhoA/ROCK inhibitors previously mentioned. The RhoA pathway is an important regulator of the cytoskeleton and cell polarity; it determines migration, proliferation, and survival. The RhoA/ROCK pathway is intimately involved with SCI in multiple ways, including apoptosis, integrity, axon regeneration, neurogenesis, and angiogenesis. Previous publications have focused on the relationship between the RhoA/ROCK pathway and apoptosis, nervous system inflammation, oxidative stress, and axon regeneration. Future studies are expected to include their effects on astrogliosis, neurogenesis, and angiogenesis.

A review on the activity of RhoA inhibitors in SCI was recently published [86]. The conclusions of the review clearly indicated that inhibitors could promote axon sprout formation and nerve fiber regeneration after SCI. They may also protect the white matter and thus the regeneration of locomotor function. According to the authors, these are the drugs that may soon become the lead structures for the clinical treatment of SCI. Further articles on this topic have been published in 2021 [87,88]. RhoA inhibitors are potential agents for promoting plasticity and neuronal growth after SCI. Significant progress has been made in recent years in the development of novel lead structures. Many studies are now in the clinical/preclinical phase, which will exactly confirm or deny the efficacy of such derivatives [89,90,91,92]. 

### 5.5. Therapy Based on the mTOR Pathway

The mTOR pathway may be another important therapeutic pathway in SCI. A review paper was published in 2021 summarizing researchers’ findings on this topic so far [93]. mTOR is a serine/threonine protein kinase that is responsible for a signaling network and is a central controller of cell growth. In addition, it plays a key role in regulating cell metabolism through its activity in translation, ribosomal biogenesis, and autophagy. Inhibition of mTOR can induce neuroprotection in multiple ways. Inhibition of mTOR can increase mitochondrial clearance through induction of autophagy, decrease cytochrome C release, and affect cell apoptosis. Inhibition of mTOR has been proven to contribute to axon regeneration and promote regeneration of nerve function and glial scarring. The role of mTOR in SCI is controversial. The majority of studies have shown that activation of the pathway does not promote regeneration, so inhibitors are a desirable entity to analyze. Inhibition of mTOR is thought to alleviate SCI and promote axonal regeneration and restoration of neurological function [93,94,95,96]. One group of compounds raising hope in the treatment of secondary SCI may be monoclonal antibodies. Although opinions and research are extreme, more attempts are still being made to study substances in this group. According to reports from the scientific world, high-mobility group box 1 (HMGB1) is a molecular pattern associated with damage and could play an important role in the treatment of SCI. An anti-HMGB1 antibody was used during a scientific study. There was a simultaneous correlation with epotilone (Epo B), which is a known anticancer drug; the substances were correlated to increase activity [97]. It has been shown that Epo B binds to B-tubulin subunits in microtubules, increases their stability, and affects cell division, migration, and neurite proliferation. Administration of low doses of Epo B promoted axon elongation, resulting in improved motor function [98]. The combination of Epo B and anti-HMGB1 was also found to be beneficial in SCIs; there was axon growth and improved locomotion. The ablation experiment confirmed that surviving intraspinal neurons are crucial for restoring function. 

Bhowmick and Abdul-Muneer conducted a study on the effect of blocking PTEN, which would promote the functional recovery of SCI. PTEN is a critical intraneuronal factor that controls the regenerative capacity of damaged CNS axons. It is a negative regulator of the Pi3K/AKT-mTOR pathway, which has an important function in controlling cell growth. A study in a rat model showed that a peptide antagonistic to PTEN promoted axon growth and behavioral regeneration. An antagonist injected 2 days after SCI may provide a basis for achieving effective axonal regeneration given how broad the Food and Drug Agency (FDA)-approved peptide drug base is in humans. Regeneration of higher density CST fibers and descending serotonergic axon fibers has been detected in mice treated in this manner [99].

Administration of a stimulating factor to reactive astrocytes with stem cell potential for oligodendrocytes is also another proposed method [100]. Neuregulin-1 (Nrg1) is a factor that plays an essential role in oligodendrocyte differentiation. The study by Zhenfei Ding et al. used adult male rats subjected to a model SCI. The injury model was established using Allen’s method, and the animals were subjected to a T9-T10 laminectomy. At the same time, an immunochemical study and a test for the potential cytotoxicity of Nrg-1 were performed. It was shown that in SCI rats, intrathecal administration of the drug converted reactive astrocytes into oligodendrocyte lineage cells, inhibited astrogliosis, promoted remyelination, protected axons, and improved the motor functions of the test animals. Motor function was assessed by two independent investigators. The biological effects were reversed when the study drug and ErbB (family of proteins containing four receptor tyrosine kinases) were co-administered, suggesting that the receptor is the drug’s point of attachment. The findings showed that Nrg1 is sufficient to differentiate reactive astrocytes into oligodendrocytes via the PI3K-AKT-mTOR signaling pathway. Promoting oligodendrocyte differentiation and proliferation has a great impact on the remyelination and recovery of SCI animals. 

### 5.6. Therapy at the Sigma-1 Receptor

Substances acting within the sigma-1 receptor have emerged as a potential therapeutic target for the treatment of secondary SCI [101]. This receptor is highly expressed within motor neurons, the loss of which is one of the major problems in SCIs. In a rat study model, a neuroprotective effect was demonstrated, where ligands of the sigma-1 receptor reduced the loss of motor neurons by approximately 20%. This process was associated with a modulation of endoplasmic reticulum stress markers. Neuroprotective effects were exerted by preventing cell death through maintenance of the IRE1/XBP1s axis. The number of surviving neurons increased, and the glial reactivity decreased in the ventral horn after rhizotomy [101,102].

### 5.7. Other Possible Drugs

Minocycline—Jiancheng Xu et al. [103] studied the effect of minocycline on neuroregeneration. In a rat animal model, the subjects were intraperitoneally injected with minocycline (50 mg/kg) 1 h after SCI, and this treatment was repeated for 5 days. In this study, it was proven that minocycline can promote BDNF (brain-derived neurotrophic factor) expression and reduce neural cell lesions. At the same time, it provides a better environment for axon regeneration.

Midostaurin—a study in 2021 was designed to investigate the effects of midostaurin on the course of secondary SCI [104]. Midostaurin is a protein kinase inhibitor. This drug was administered via an intraperitoneal injection of 25 mg/kg body weight to rats with SCI in the C6–C7 region. Motor function, as in previous studies, was assessed by limb grip strength. A histopathological analysis was also performed. The drug was shown to alter local inflammation. The treated animals showed increased recovery and coordination between the hind and fore limbs. Both results indicate a synergistic effect of the test chemical in improving functional regeneration. After the histopathological analysis, it was found that tissue functionality was significantly increased. This may be an effective strategy for alleviating secondary SCI.

Resolvin—Juri Kim et al. [105] have recently demonstrated the neuroprotective effect of Resolvin D3. This compound belongs to the Resolvin group, a family of endogenous mediators that promote the cessation of the inflammatory response. The effects of anti-inflammatory and neuroprotective activity were tested in a mouse animal model and in vivo on RAW 264.7 cells and the human brain endothelial cell line hCMEC/D3. Female mice after SCI received an intrathecal injection of RvD3 vehicle at a dose of 1 ug/20 uL 1 h after surgery. RvD3 reduced the production of inflammatory mediators and nitric oxide in RAW 264.7 cells and promoted angiogenic effects in the hCMEC/D3 cell line. In the study practice, the drug improved recovery and reduced patients’ hypersensitivity. The production of TNF-alpha, IL-6, IL-f1B, CCL2, and CCL2 was also reduced. Immunohistochemistry showed decreased gliosis and neuroinflammation and neuroprotection.

Lithium chloride—a recent study examined the effect of lithium chloride on SCI at the T10 level in adult male rats [106]. The Basso–Beattie–Bresnahan locomotor rating scale (BBB) test was used to evaluate the degree of function. One day after surgery, animals in the study sample were intraperitoneally given lithium chloride at a dose of 85 mg/kg. Animals with SCI were retested at the BBB by two independent researchers on a scale ranging from 0 to 21. After killing the animals, a histopathological examination of the core was performed. It was found that administration of lithium chloride alleviated pathological SCI and inhibited TNF-alpha, IL-6, and IL-1B. At the same time, pyroptosis was inhibited, and inflammation and oxidative stress were reduced. The neuroprotective effect of lithium on anaerobic PC12 cells (dopaminergic lineage) was also investigated. Lithium decreased apoptosis and upregulated nuclear factor erythroid 2-related factor 2 (Nrf2) and heme oxygenase-1 in PC-12 cells. In addition, an Nrf2 inhibitor was shown to reverse the effects of lithium. On this basis, it can be concluded that the test substance acts through the Nrf2/hemoxygenase-1 pathway, promotes recovery after SCI, and has a neuroprotective effect.

Enoxaparin—the last study on the topic of neuroprotection that we wish to highlight is the effect of enoxaparin on SCI functional recovery. Enoxaparin is a well-known anticoagulant used in pulmonary embolic disease and deep vein thrombosis. The effect of this substance on receptor-type protein tyrosine phosphatase (PTPRq) was investigated in a scientific study [67]. This is a regulatory unit for axon sprouting. As in previous studies, SCI at the T9 level was reproduced. The injury completely destroyed the dorsal columns and the dorsal corticospinal tract. The animals in the study group were subcutaneously administered 50 ug/day of enoxaparin, and motor function was assessed by the BBB and foot defect tests. The recovery of sensory function analyzed by the tactile test was significantly better than that of the control group. Mechanical allodynia was alleviated. It was proven that the minimum effective treatment time was at least 3 weeks. At the same time, no therapeutic effect of enoxaparin was observed in more chronic conditions when treatment was started after 1 and 4 weeks of SCI. The BBB score was significantly higher in the study group. The number of foot defects was also significantly lower at both 56- and 84-days post-injury. It was concluded that enoxaparin promoted electrophysiological recovery after SCI. Enoxaparin showed strong interaction with PTPRq at high stoichiometry in vitro. In comparison, fondaparinux (a comparator substance) showed a very weak interaction with PTPRq. It was emphasized that the results for enoxaparin are favorable, but further analyses are needed [107].

## 6. Spasticity and Muscle Loss

Patients suffering from SCI struggle with many obstacles during daily life activities. One of the factors reducing mobility of this group of patients is volumetric muscle loss. Both nutritional supplementation and rehabilitation therapy are beneficial in muscle reconstruction [108]. Treatment with anabolic agents such as androgens and myostatin inhibitors has a favorable impact on the inhibition of muscle atrophy; however, better results are observed during ursolic acid and beta2-agonists usage [109].

SCI might be manifested by osteoporosis. Unfortunately, for now there is a lack of screening and comprehensive guidelines for patients with SCI that are experiencing sublesional bone loss. Hence, the ability of treatment is limited to the infrequent use of supplements—especially vitamin D—and physical activity [110]. A danger for patients with SCI and osteoporosis is fragility fracture. The rate related to fragility fracture in this group of patients is twice that of the general population. However, factors influencing this rate are unknown, and more research is needed [111]. Among a trial group of 20 patients who experienced bone loss after SCI, the most effective intervention was longer testosterone treatment combined with resistance training [112].

Another complication observed among patients after SCI is spasticity; at present, there is no sufficient form of treatment for this symptom. A delay in the appearance of spasticity after an SCI is obtained by early escitalopram administration, which as a selective serotonin reuptake inhibitor increases serotonin (5-HT) levels, causing desensitization to 5-HT receptors increased in spasticity [113]. Research about treatment of spasticity also includes medications such as tolperisone, which is a centrally acting skeletal muscle relaxant, or baclofen-stimulating GABA beta receptors. Administration of these drugs was combined with physical therapy. In this study, several adverse effects were observed. Patients after treatment with baclofen experienced asthenia and sleepiness. On the other hand, tolperisone caused dyspepsia and epigastric pain [114]. According to the studies, one of the adverse effects of baclofen observed among animals might be cognitive impairment, especially combined with the memory. Nevertheless, research from 2021 on 22 patients with SCI treated with baclofen revealed different findings. Assessment of the cognitive function of these trial patients revealed no decline in memory functions; an improvement in short-term auditory-verbal memory and logical memory performance was even observed [115]. Based on the case of a 53-year-old male suffering from SCI and who was treated with baclofen for 14 years, the management of autonomic dysreflexia (AD) might be categorized as a potential action of this drug; however, further studies are necessary. Treatment of the featured patient due to an abscess in the pump pocket was gradually reduced to prevent a withdrawal syndrome. After the pump was explanted, the patient experienced severe AD, which was successfully controlled after baclofen reapplication [116].

The only pharmacological treatment for the spasticity of strokes and cerebral palsy is botulinum toxin, which is produced by the bacterium *Clostridium botulinum* [117]. Through binding to high-affinity recognition sites on the cholinergic nerve terminal, botulinum toxin decreases the release of acetylcholine and causes neuromuscular blocking. Botulinum toxin is used in treatment for focal spasticity too. It should also be added that although treatment with botulinum toxin reduces spasticity, an improvement in voluntary movement is not observed [118]. Intramuscular injections of botulinum toxin may cause myositis, which was observed in a 17-year-old male suffering from spasticity after SCI who was treated with botulinum toxin [119]. Moreover, constipations due to spasticity developed after an SCI might be treated using botulinum toxin. In this situation, botulinum toxin administration to the external anal sphincter causes an alleviation of constipation symptoms [118]. Another complication observed in a group of patients after SCI is neurogenic detrusor overactivity, which leads to urinary incontinence. The investigation conducted on 61 patients suggested an advantageous impact of botulinum toxin treatment for the patients’ urodynamic parameters [120]. Spasticity developed after SCI might occur in abdominal muscles. Botulinum toxin injection for the bilateral internal oblique and external oblique abdominal muscles with ultrasonography guidance was an alternative form of treatment for a patient who was unsuccessfully cured when treated with oral baclofen [121].

Furthermore, to aim for an effective treatment for spasticity, researchers are attempting to efficiently differentiate transplant human pluripotent stem cells from spinal GABA neurons [122] and aim to transplant peripheral nerves with long-term infusions of fibroblast growth factor [123]. So far, the examinations have been conducted among animals, but their results are promising. 

## 7. Conclusions

The above publication analyzed dozens of studies on SCI published within the last year. They have been divided into main chapters according to the health problems of patients with SCI. This publication is a compendium of contemporary pharmaceutical care for patients with SCI and is a review of the latest medical discoveries in secondary SCI therapy. It can be a starting point for researchers creating new paths of research related to the CNS. At the same time, the enrichment of knowledge in the above-mentioned topic may inspire and contribute to the validation of existing scientific theories, which will ultimately improve the quality and length of life of patients struggling with tetraplegia and paraplegia.

## Data Availability

Not applicable.

## List of Abbreviations

ABRT-scES	Activity-based recovery training epidural stimulation of the spinal cord
BTX-A	Botulinum toxin A
ChABC	Chondroitinase ABC enzyme
CNS	Central nervous system
CSPG	Chondroitin sulfate proteoglycans
EAS	External anal sphincter
ENSC	Intestinal neutral stem cells
hIL6	Fusion protein of the cytokine interleukin-6 and the soluble interleukin-6 receptor, which are covalently linked by a flexible peptide linker
LUT	Functional impairment of the lower urinary tract
LV	Lentiwirus
mTOR kinase	Threonine-serine protein kinase, whose function is to regulate cell growth, proliferation, and movement, as well as translation and transcription processes
NDO	Detrusor neurogenic hyperactivity
OAB	Overactive bladder
onaBoNTA	OnabotulinumtoxinA
PC protein	Biochemically serine proteinase is a physiological blood anticoagulant, inactivating active factors VIIIa and Va and thus inhibiting thrombin and clot fibril formation
PTEN	The PTEN gene belongs to the suppressor genes, which means that its protein product is involved in cell cycle regulation
SCC	Spinal cord crush
scES	Epidural stimulation of the spinal cord
SCI	Spinal cord injury
TNF-alpha	Tumor necrosis factor alpha
TrkB, 29D7	Antibody that binds to the TRKB receptor, matched by a key-clamp model
TRKD receptor	Tropomyosin B receptor kinase
